# Maternal and neonatal outcomes in obstetric antiphospholipid syndrome: a retrospective case-control study

**DOI:** 10.3389/fmed.2025.1660134

**Published:** 2025-12-10

**Authors:** Lin Rao, Jia Lu, Hong Li, Liang Xu, Dongjian Yang, Wendong Han, Li Chen

**Affiliations:** 1 Key Laboratory of Medical Molecular Virology (MOE/NHC/CAMS), School of Basic Medical Sciences, Shanghai Medical College, Fudan University, Shanghai, China; 2 Department of Nursing, International Peace Maternity and Child Health Hospital, Shanghai Jiao Tong University School of Medicine, Shanghai, China; 3 Key Laboratory of Embryo Original Diseases, Shanghai, China; 4 Department of Gynaecology, International Peace Maternity and Child Health Hospital, Shanghai Jiao Tong University School of Medicine, Shanghai, China; 5 Xinhua Hospital Affiliated to Shanghai Jiao Tong University School of Medicine, Shanghai, China; 6 Clinical Research Center, International Peace Maternity and Child Health Hospital, Shanghai Jiao Tong University School of Medicine, Shanghai, China; 7 Shanghai Institute of Virology, Shanghai Jiao Tong University School of Medicine, Shanghai, China

**Keywords:** obstetric antiphospholipid syndrome (OAPS), placental vasculopathy, non-canonical antiphospholipid antibodies, neonatal hyperbilirubinemia, pregnancy-related hypercoagulability, multidisciplinary perinatal management

## Abstract

**Objective:**

The combination of low-dose aspirin (LDA) and low-molecular-weight heparin (LMWH) is the standard of care for obstetric antiphospholipid syndrome (OAPS), significantly improving live birth rates. However, whether this regimen fully normalizes the pregnancy course and mitigates risks for both the mother and the neonate remains unclear. This study aimed to systematically evaluate whether significant maternal and neonatal morbidity persists in OAPS patients despite successful treatment and live birth.

**Methods:**

This retrospective cohort study included 256 OAPS patients, including 166 criteria OAPS patients (C-OAPS—patients who fulfilled both the clinical and laboratory criteria of the Sydney criteria) and 90 non-criteria OAPS patients (NC-OAPS—patients who fulfilled only the clinical or only the laboratory criteria of the Sydney criteria) who achieved live birth, along with 768 matched healthy controls. We compared basic characteristics, laboratory parameters, and perinatal outcomes between the groups.

**Results:**

Compared to healthy controls (*n* = 768), treated OAPS patients (*n* = 256) exhibited a persistent hypercoagulable state (elevated D-dimer and fibrin degradation product (FDP), *p* < 0.01) and a higher incidence of anemia (*p* < 0.001). Their neonates had significantly lower birth weight (*p* = 0.006) and elevated risks of neonatal infection (adjusted OR = 3.12, *p* = 0.004) and hyperbilirubinemia (adjusted OR = 2.06, *p* = 0.024), with the infection risk remaining significant in full-term infants. A subgroup analysis revealed no significant differences in obstetric history, maternal complications, comorbidities, and outcomes between the C-OAPS and NC-OAPS groups.

**Conclusion:**

Despite standard treatment, OAPS patients who deliver successfully remain at an increased risk for persistent maternal hypercoagulability and adverse neonatal outcomes. These findings underscore the need for a paradigm shift in management—from merely ensuring live birth to safeguarding neonatal health through proactive, multidisciplinary perinatal care.

## 1 Introduction

Antiphospholipid syndrome (APS) is a systemic autoimmune disorder characterized by clinical manifestations, including thrombotic events and/or obstetric complications, and it is accompanied by the presence of antiphospholipid antibodies (aPLs) (1). The 2006 Sydney International Consensus provides an extensive overview of adverse obstetric outcomes linked to recurrent first-trimester miscarriage, fetal loss, stillbirth, early and severe pre-eclampsia, or preterm birth (before 34 weeks of gestation) (2). In patients with a history of thrombosis, these complications are classified as obstetric APS (OAPS) and are primarily attributed to placental dysfunction (3). This is partly due to the detrimental effects of aPLs throughout the stages from implantation and placentation to delivery (4).

Recent research has indicated that placental inflammatory responses, including complement activation and subsequent endothelial damage in patients with OAPS, may impair the invasive function of placental trophoblasts (5). Additionally, the formation of neutrophil extracellular traps (6), release of interleukin-8 (7), upregulation of the target of rapamycin complex on endothelial cells (8), and an imbalance in angiogenic factors (9) do not culminate in thrombosis. While the administration of glucocorticoids appears promising in mitigating inflammatory response (10), the current gold standard in clinical treatment remains the combination of low-dose aspirin (LDA) and low-molecular-weight heparin (LMWH) (11). This treatment regimen has significantly increased the rate of successful deliveries in patients with OAPS; however, potential maternal placental pathology and effects on the offspring cannot be ruled out, due to the persistence of aPLs.

However, whether this treatment fully normalizes the course of pregnancy and eliminates risks for both the mother and neonate remains a subject of inquiry. A focus solely on live birth may overlook significant maternal and neonatal morbidity that persists despite treatment. For instance, some reports suggest an association between OAPS and adverse outcomes such as fetal growth restriction, preterm birth, and placental pathology even in treated pregnancies (9). Furthermore, a notable proportion of patients with clinical features highly suggestive of OAPS tested negative for the criteria OAPS (C-OAPS), a category often referred to as seronegative or non-criteria OAPS (NC-OAPS) (12), presenting a diagnostic and management challenge. According to the 2023 American College of Rheumatology (ACR)/European Alliance of Associations for Rheumatology (EULAR) classification criteria for antiphospholipid syndrome, these patients are likely to be classified into an under-recognized group. Although the new standards significantly enhance specificity and introduce a more detailed clinical and laboratory stratification weighting system, their strict inclusion criteria—requiring a cumulative score of at least 3 in both clinical and laboratory domains—may result in the exclusion of some patients with typical obstetric clinical manifestations whose laboratory tests do not meet the threshold from the classification (13).

Given these considerations, this study was conducted to systematically evaluate the pregnancy characteristics and outcomes in a cohort of OAPS patients who achieved a live birth following standard treatment. We aimed to retrospectively analyze and compare basic maternal characteristics, laboratory parameters, and perinatal outcomes between these treated OAPS patients and matched healthy controls. The objective of this study was to assess the potential persistent effects of OAPS on pregnancy and to provide a detailed clinical profile that may inform future management strategies and research directions.

## 2 Materials and methods

### 2.1 Study population

**Figure 1:**
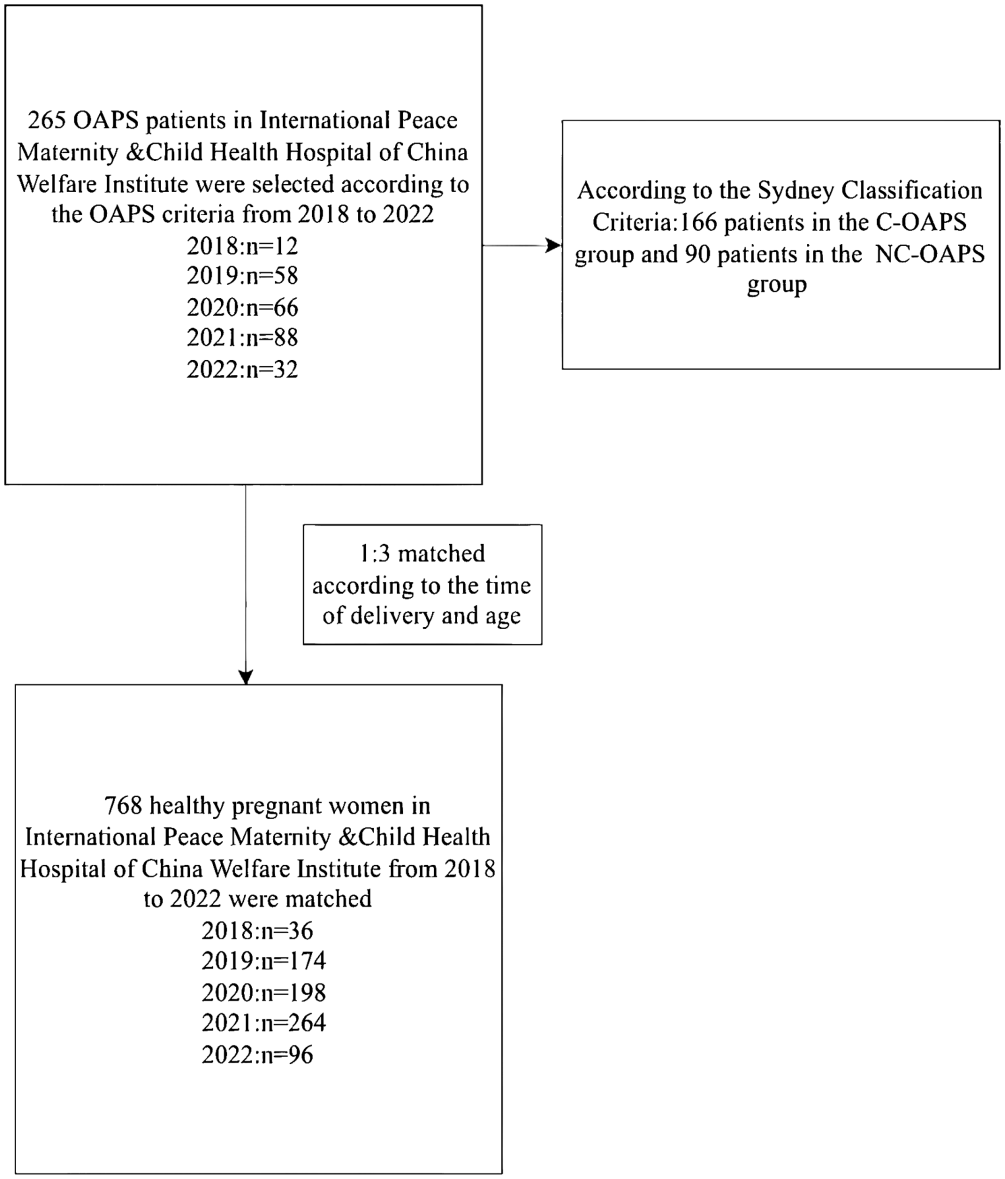
Study population.

All medical histories for this study were recorded at the International Peace Maternity and Child Health Hospital of the China Welfare Institute from 2018 to 2022. All research participants had delivered successfully (see Figure 1).

### 2.2 The inclusion and exclusion criteria of the patients with OAPS

#### 2.2.1 Clinical inclusion criteria for C-OAPS and NC-OAPS

Patients with OAPS are classified as either the C-OAPS group or the NC-OAPS group according to the Sydney criteria.

C-OAPS required at least one clinical criterion plus one laboratory criterion. Conversely, those who fulfilled the criteria in only one aspect—that is, patients who fulfilled the clinical criteria but did not fulfill the laboratory criteria or those who fulfilled the laboratory criteria but did not fulfill the clinical criteria—were classified as having non-criteria OAPS (NC-OAPS) (14). In accordance with the prevailing clinical guidelines, all enrolled OAPS patients were managed with a combination of low-dose aspirin and prophylactic low-molecular-weight heparin throughout pregnancy (from the first confirmation of pregnancy until at least 6 weeks postpartum) to improve obstetric outcomes (15).

(1) The clinical criteria were unexplained fetal death ≥10 weeks, premature birth <34 weeks due to placental dysfunction, or ≥3 consecutive miscarriages <10 weeks.
(2) The laboratory criteria were lupus anticoagulant (LA), medium−/high-titer immunoglobulin G (IgG)/immunoglobulin M (IgM) anticardiolipin (aCL), or anti-β2-glycoprotein-I (anti-β2GPI) antibodies—mandated positivity on two occasions at least 12 weeks apart.

#### 2.2.2 Exclusion criteria

The clinical exclusion criteria were as follows:

(1) Patients with active hepatitis B virus, hepatitis C virus, human immunodeficiency virus, syphilis, or tuberculosis infection;
(2) Those with a history of smoking or drinking;
(3) Those who use drugs during pregnancy which can seriously affect maternal and infant outcomes;
(4) Those who underwent induced abortion due to family planning or personal request;
(5) Those with a history of significant diseases, such as severe lesions of vital organs, during pregnancy;
(6) Those with a history of previous thrombosis or pregnancy complicated by thrombosis;
(7) Those without routine prenatal examinations and whose data were incomplete; and
(8) Those with multiple gestations.

### 2.3 The inclusion and exclusion criteria of the healthy pregnant women

Case and control groups were matched at a 1:3 ratio according to the time of delivery and age to reduce confounding factors and selection bias.

#### 2.3.1 Inclusion criteria for healthy pregnant women

The inclusion criteria for healthy pregnant women were as follows:

(1) Previously healthy pregnant women who gave birth during the same period as the case group;
(2) Women who did not meet any of the OAPS and non-criteria OAPS diagnostic criteria;
(3) Those with no history or family history of autoimmune disease; and
(4) Those who have not used drugs such as corticosteroids and immunosuppressants.

#### 2.3.2 Exclusion criteria for healthy pregnant women

The exclusion criteria were the same as the OAPS patients.

### 2.4 Clinical data collection

Our study collected three types of information: basic information, laboratory examinations, and clinical characteristics during the perinatal period.

#### 2.4.1 Basic information

Basic information included age, pre-pregnancy body mass index (BMI), weight gain during pregnancy, days of latest pregnancy, number of deliveries, number of pregnancies, history of preterm labor, history of stillbirth, and number of miscarriages.

#### 2.4.2 Laboratory examination

Upon admission to the hospital for delivery, blood samples were collected. Relevant experimental indices, including international normalized ratio (INR), activated partial thromboplastin time (APTT), thromboplastin time (TT), prothrombin time (PT), D-dimer, FDP, total bile acid (TBA), red blood cells (RBCs), hemoglobin (HB), and platelets (PLTs), were tested.

#### 2.4.3 Detection of antiphospholipid antibodies: main instruments and reagents

Our laboratory used the enzyme-linked immunosorbent assay (ELISA) as the core detection technology, utilizing the fully automated EUROIMMUN analyzer and corresponding test kits to perform quantitative detection of antiphospholipid antibodies in clinical serum samples.

##### 2.4.3.1 Main instruments

The main instruments were the Fully Automated Fluorescence Immunoassay Analyzer SPRINTER XL from EUROIMMUN.

##### 2.4.3.2 Reagent kits (all from EUROIMMUN)

The reagent kits included the Anti-Cardiolipin Antibody IgA Test Kit (product code: EA 1621-9601A), Anti-Cardiolipin Antibody IgG Test Kit (product code: EA 1621-9601G), Anti-Cardiolipin Antibody IgM Test Kit (product code: EA 1621-9601 M), Anti-β2-Glycoprotein I Antibody IgG Test Kit (product code: EA 1632-9601G), and Anti-β2-Glycoprotein I Antibody IgM Test Kit (product code: EA 1632-9601 M).

#### 2.4.4 Clinical characteristics of the perinatal period

To accurately gauge the severity of obstetric complications, the study adhered to the Sydney International Consensus, which categorizes complications into three primary groups:

(1) Adverse pregnancy outcomes: intrauterine distress, fetal growth restriction (FGR), and postpartum hemorrhage;
(2) Maternal complications and comorbidities: diabetes mellitus in pregnancy (pre-pregnancy diabetes mellitus and gestational diabetes mellitus [GDM]), hypertensive disorders of pregnancy (gestational hypertension [GH] and pre-eclampsia/eclampsia), thyroid disorders of pregnancy, intrahepatic cholestasis in pregnancy, hematologic disorders of pregnancy (anemia), placental abnormalities (placental adhesion, placental lakes, placental abruption, and placental implantation), abnormalities (abnormal amount and color of amniotic fluid), pregnancy-related sexually transmitted diseases (mycoplasma infection), and pregnancy-related streptococcal infection;
(3) Neonatal complications: preterm labor, low birth weight, neonatal asphyxia, neonatal infections, and neonatal hyperbilirubinemia.

### 2.5 Statistical methods

Values are expressed as mean (±S.D.) and numbers and percentages for qualitative variables. Student’s t-test was used to compare continuous variable data following a normal distribution, while Mann–Whitney Wilcoxon’s test was used for continuous variable data not following a normal distribution. The chi-squared test, Yates’s correction for continuity, and Fisher’s exact test were applied to compare categorical variables. For comparisons of continuous variables that were not normally distributed across the three independent groups, the Kruskal–Wallis H test was used. Post-hoc pairwise comparisons were then conducted using Dunn’s test, applying a Bonferroni correction for multiple comparisons. A difference was considered statistically significant at a *p*-value of <0.05. To further evaluate the independent association between obstetric antiphospholipid syndrome (OAPS) and maternal/neonatal outcomes, binary logistic regression analyses were performed. Outcomes that showed significant differences (*p* < 0.05) in the univariate analyses were included in the multivariate models. The regression models were adjusted for potential confounding factors identified from clinical relevance and univariate results; the results are expressed as adjusted odds ratios (ORs) with 95% confidence intervals (CIs). The statistical software SPSS 23.0 (IBM, United States) was used for dataset analyses.

## 3 Results

A total of 1,024 women were included in this study: 256 OAPS cases and 768 healthy controls (HCs). The OAPS group was divided into 166 patients in the typical OAPS group and 90 patients in the atypical NC-OAPS group, according to the Sydney Classification Criteria. All of the participating women had delivered successfully following treatment during the most recent pregnancy in the hospital. We also randomly selected 768 healthy pregnant women admitted to the obstetrics department during the same period, who were matched at a 1:3 ratio to the OAPS group based on the time of delivery and age.

### 3.1 Basic information and reproductive history of the OAPS and healthy control groups

In this study, significant differences were observed in gestational weight gain and gestational duration between the OAPS and HC groups. The OAPS group exhibited a higher mean weight gain (12.35 ± 5.69 kg) than the HC group (10.35 ± 7.10 kg, *p* < 0.001), as well as a shorter mean gestational period (266.38 ± 9.85 days versus 270.44 ± 10.98 days, *p* < 0.05). Additionally, marked disparities in gravidity and parity were evident between the groups. Compared to the HC group (32.81%), the OAPS group had a higher incidence of pregnancies with three or more instances of gravidity (64.06%) and a lower incidence of two or more instances of parity (21.87% versus 29.30%). Furthermore, a significant difference was observed in adverse maternal history: the incidence of preterm deliveries was substantially higher in the OAPS group (10.55%) than in the HC group (0.26%), and the rate of stillbirths was higher in the OAPS group (6.64%) than in the HC group (1.04%). Moreover, the OAPS group exhibited a higher frequency of miscarriages, with two and three or more occurrences corresponding to percentages of 37.11% versus 16.15 and 24.61% versus 8.20%, respectively. There were no differences between the C-OAPS and NC-OAPS groups (refer to Table 4).

**Table 4:** Basic information and obstetric history of the OAPS group and the healthy control group.

Characteristic	OAPS	C-OAPS (*n* = 166)	NC-OAPS (*n* = 90)	HC (*n* = 768)	t/t’/z/𝑥^2^ (OAPS vs. HC)	*p-*value (OAPS vs. HC)	z/𝑥^2^/H (C-OAPS vs. NC-OAPS vs. HC)	*p-*value (C-OAPS vs. NC-OAPS vs. HC)
Age (years)	32.73 ± 4.08	32.61 ± 4.14	32.96 ± 3.98	32.27 ± 3.18	−1.620	0.148	2.565	0.277
Pre-pregnancy BMI	22.15 ± 3.22	22.31 ± 3.37	21.87 ± 2.92	21.82 ± 3.11	−1.448	0.526	0.905	0.636
WG during pregnancy	12.35 ± 5.69	12.03 ± 6.00^a^	12.94 ± 5.08^a^	10.35 ± 7.10^b^	−3.990	<0.001**	10.41	0.005*
Gestation (days)	266.38 ± 9.85	265.53 ± 10.21^a^	267.93 ± 8.99^a^	270.44 ± 10.98^b^	4.397	0.036*	51.04	<0.001**
Gravidity								
1	36 (14.06%)	28 (16.87)^a^	8 (8.89)^a^	311 (40.50%)^b^	87.488	<0.001**	90.473	<0.001**
2	56 (21.88%)	38 (22.89)^a^	18 (20.00)^a^	205 (26.69%)^a^				
≥3	164 (64.06%)	100 (60.24)^a^	64 (71.11)^a^	252 (32.81%)^b^				
Parity								
1	200 (78.13%)	132 (79.52)	68 (75.56)	543 (70.70%)	5.312	0.021*	5.772	0.056
≥2	56 (21.87%)	34 (20.48)	22 (24.44)	225 (29.30%)				
History of adverse maternal outcomes								
Preterm labor	27 (10.55%)	18 (10.84)	9 (10.00)	2 (0.26%)	70.140^#^	<0.001**	63.629^#^	<0.001**
Stillbirth	17 (6.64%)	14 (8.43)	3 (3.33)	8 (1.04%)	25.270	<0.001**	23.736^#^	<0.001**
Number of miscarriages								
1	57 (22.27%)	38 (22.89)^a^	19 (21.11)^a^	193 (25.13%)^a^	5.903	0.015*	0.954	0.621
2	95 (37.11%)	58 (34.94)^a^	37 (41.11)^a^	124 (16.15%)^b^	128.349	<0.001**	51.509	<0.001**
≥3	63 (24.61%)	38 (22.89)^a^	25 (27.78)^a^	63 (8.20%)^b^	103.507	<0.001**	49.184	<0.001**

OAPS, obstetric antiphospholipid syndrome; HC, health control; BMI, body mass index; WG, weight gain. ^#^, Yates’s correction for continuity; *, *p* < 0.05; **, *p* < 0.001. Values are significantly different within a column followed by different lowercase letters.

### 3.2 Results of laboratory test parameters

The incidence of APTT and PT abnormalities was significantly greater in the OAPS group than in the HC group (*p* < 0.001). Conversely, the incidence of PTA abnormalities was lower in the OAPS group than in the HC group (*p* = 0.023). Additionally, patients with OAPS exhibited higher rates of abnormalities in D-dimer, FDP, and TBA than the HC group (*p* < 0.001), with abnormality rates of 14.51% vs. 7.42, 13.67% vs. 3.91, and 3.52% vs. 0.39%, respectively. Furthermore, the incidence of RBC abnormalities was higher in the OAPS group than in the HC group (*p* = 0.02), consistent with the trend observed in the HB test (*p* = 0.001) (see Table 1).

**Table 1:** Results of laboratory test parameters.

Parameters	Normal range	Proportion of patients with abnormal laboratory findings			
		OAPS group *n* (%)	HC group *n* (%)	𝑥^2^	*p-*value
INR	0.75–1.25	0 (0.00)	0 (0.00)	/	/
APTT	28–45 s	61 (23.83)	3 (0.39)	178.89^#^	<0.001^***^
TT	14-21 s	6 (2.34)	3 (0.39)	0.034^#^	0.854
PT	11-14.5 s	47 (18.36)	1 (0.13)	141.92^#^	<0.001^***^
PTA	2–4 g/L	184 (71.88)	602 (78.38)	5.172	0.023^*^
D-Dimer	0–3.05 mg/L	37 (14.45)	57 (7.42)	11.30	0.001^**^
FDP	<10 mg/L	35 (13.67)	30 (3.91)	28.419	<0.001^***^
TBA	0–10 μmol/L	9 (3.52)	3 (0.39)	13.60^#^	<0.001^***^
RBC	3.68–5.13 × 10^12^/L	91 (35.55)	211 (27.47)	5.44	0.02^*^
HB	113–151 g/L	93 (36.33)	194 (25.26)	10.867	0.001^**^
PLT	101–320 × 10^9^/L	16 (6.25)	46 (5.99)	0.01	0.917

INR, international normalized ratio; APTT, activated partial thromboplastin time; PT, prothrombin time; FDP, fibrin degradation product; TBA, total bile acid; RBC, red blood cells; HB, hemoglobin; PLT, platelet; OAPS, obstetric antiphospholipid syndrome; HC, healthy controls. ^#^, Yates’s correction for continuity; ^*^, *p* < 0.05; ^**^, *p* < 0.01; ^***^, *p* < 0.001.

### 3.3 Maternal complications, comorbidities, and outcomes

The prevalence of anemia was significantly greater in the OAPS group than in the HC group (*p* < 0.001). Furthermore, no significant difference was observed in the incidence of abnormal placental invasion and morphological anomalies between the OAPS group and the HC group (*p* > 0.05). Additionally, the rate of cesarean delivery was higher in the OAPS group than in the HC group (*p* = 0.048). Among the pregnancy outcomes, neonatal weight was significantly lower in the OAPS group than in the HC group (*p* = 0.006). Conversely, the incidence of intrauterine distress was significantly higher in the HC group than in the OAPS group (*p* = 0.001). There were no differences between the C-OAPS and NC-OAPS groups (Table 2).

**Table 2:** Clinical characteristics of the perinatal period.

Clinical characteristics of the perinatal period	OAPS group (*n* = 256) *n* (%)	C-OAPS (*n* = 166) *n* (%)	NC-OAPS (*n* = 90) *n* (%)	HC group (*n* = 768) *n* (%)	𝑥^2^ (OAPS vs. HC)	*p-*value (OAPS vs. HC)	𝑥^2^/H (C-OAPS vs. NC-OAPS vs. HC)	*p-*value (C-OAPS vs. NC-OAPS vs. HC)
Pregnancy complications								
GDM	66 (25.78)	45 (27.11)	21 (23.33)	169 (22.01)	1.342	0.213	2.019	0.364
TD	37 (14.45)	23 (13.86)	14 (15.56)	151 (19.66)	3.316	0.062	3.578	0.166
Anemia	68 (28.56)	45 (27.11)^a^	23 (25.56)^a^	65 (8.46)^b^	54.06	<0.001***	55.776	<0.001***
Cholestasis	4 (1.56)	3 (1.81)	1 (1.11)	9 (1.17)	0.026^#^	0.872	/	0.706^##^
GH	19 (7.42)	14 (8.43)	5 (5.56)	47 (6.12)	0.345	0.462	1.342	0.511
PE/Eclampsia	11 (4.30)	10 (6.02)	1 (1.11)	29 (3.78)	0.035	0.71	/	0.177^##^
Placental condition and abnormalities								
PAd	19 (7.42)	12 (7.23)	7 (7.78)	39 (5.08)	1.56	0.160	2.007	0.367
PL	3 (1.17)	1 (0.60)^a,b^	2 (2.22)^b^	1 (0.13)^a^	3.012^#^	0.083	/	0.017^##^*
PI	2 (0.78)	1 (0.60)	1 (1.11)	2 (0.26)	0.335^#^	0.563	/	0.262^##^
PAb	2 (0.78)	1 (0.60)	1 (1.11)	1 (0.13)	1.003^#^	0.317	/	0.156^##^
Placenta area (cm^2^)	316.97 ± 94.01	308.70 ± 84.52^a^	332.03 ± 108.09^a,b^	330.75 ± 90.55^b^	2.08	0.630	6.55	0.038*
Placenta weight (g)	617.26 ± 223.43	624.83 ± 267.74^a^	603.32 ± 99.57^a,b^	635.67 ± 190.43^b^	1.17	0.926	7.68	0.022*
Delivery mode (*n*, %)								
Vaginal delivery	213 (83.20)	133 (80.12)^a^	80 (88.89)^a,b^	676 (88.02)^b^	3.89	0.048*	7.814	0.02*
Cesarean delivery	43 (16.80)	33 (19.88)	10 (11.11)	92 (11.98)				
Thrombotic events	0	0	0	0	/	/	/	/
Pregnancy outcomes								
Neonatal weight (g)	3114.00 ± 481.71	3089.69 ± 497.48^a^	3158.84 ± 450.43^a,b^	3212.75 ± 529.6^b^	2.77	0.006**	10.961	0.004**
FGR (*n*, %)	9 (3.52)	5 (3.01)	4 (4.44)	19 (2.47)	0.78	0.38	/	0.452^##^
IUCD (*n*, %)	25 (9.76)	17 (10.24)^a^	8 (8.89)^a^	148 (19.27)^b^	12.36	0.001***	/	0.002**
Apgar score								
1 min	9.76 ± 0.79	9.69 ± 1.02	9.79 ± 0.66	9.72 ± 0.91	−0.18	0.854	0.114	0.945
5 min	9.93 ± 0.35	9.89 ± 0.53	9.97 ± 0.24	9.91 ± 0.45	−0.1	0.919	2.409	0.300
10 min	9.97 ± 0.21	9.98 ± 0.15	10	9.98 ± 0.12	−0.64	0.519	2.087	0.352
IBL (mL)	291.89 ± 175.80	298.21 ± 238.06	281.69 ± 161.11	292.40 ± 213.99	−0.038	0.436	1.799	0.407
PPH (*n*, %)	8 (3.13)	5 (3.01)	3 (3.33)	28 (3.65)	0.154	0.695	/	0.957^##^
SPPH (*n*, %)	5 (1.95)	3 (1.81)	2 (2.22)	6 (0.78)	1.501^#^	0.221	/	0.146^##^

GDM, gestational diabetes mellitus; TD, thyroid disease; GH, gestational hypertension; PE, pre-eclampsia; PAd, placental adhesion; PL, placental lakes; PAb, placental abruption; PI, placental implantation; OAPS, obstetric antiphospholipid syndrome; HC, healthy controls; FGR, fetal growth restriction; IUCD, intrauterine distress; IBL, intrapartum blood loss; PPH, postpartum hemorrhage; SPPH, severe postpartum hemorrhage; OAPS, obstetric antiphospholipid syndrome; HC, healthy controls. Fetal growth restriction (FGR) is defined as fetal growth that does not reach its genetic potential due to maternal, fetal, placental, and other pathologic factors, most often identified using fetal ultrasound-estimated weight or abdominal circumference below the 10th percentile of the corresponding gestational age. Postpartum hemorrhage (PPH) is defined as bleeding greater than or equal to 500 mL in vaginal deliveries and 1,000 mL in cesarean deliveries within 24 h after the delivery of the fetus or loss of blood accompanied by symptoms or signs of hypovolemia. Severe postpartum hemorrhage (SPPH) is defined as bleeding greater than or equal to 1,000 mL within 24 h after delivery. ^#^, Yates’s correction for continuity; ^##^, Fisher’s exact test; ^*^, *p* < 0.05; ^**^, *p* < 0.01; ^***^, *p* < 0.001. Values are significantly different within a column followed by different lowercase letters.

The analysis demonstrated that there was no statistically significant difference in the percentage of preterm neonates with low birth weight between the two groups (*p* = 0.893). However, the incidence of low birth weight was significantly higher among preterm neonates in the OAPS group than in the HC group (*p* = 0.004), while it was significantly lower among full-term neonates (*p* < 0.001). Regarding neonatal complications, the findings indicated that both the incidence of neonatal infections and the prevalence of neonatal hyperbilirubinemia were significantly higher in the OAPS group than in the HC group (*p* = 0.004, *p* = 0.024). For full-term infants, the incidence of neonatal infection and neonatal asphyxia was significantly greater in the OAPS group than in the HC group (*p* = 0.013, *p* = 0.011). Additionally, the occurrence of complications, including infection, asphyxia, and hyperbilirubinemia, was markedly higher in preterm infants in the OAPS group than in those in the HC group (*p* < 0.001) (refer to Table 3). There were no differences between the C-OAPS and NC-OAPS groups (Table 2).

**Table 3:** Neonatal complications among all neonates and by gestational age at birth.

Neonatal complications	OAPS *N* = 256 (*n*, %)	C-OAPS *N* = 166 (*n*, %)	NC-OAPS *N* = 90 (*n*, %)	HCN *N* = 768 (*n*, %)	*p*-value (χ2) (OAPS vs HC)	*p*-value (logistic) (OAPS vs HC)	OR (95%CI)
All neonates							
LBWI	21(8.20)	14 (8.43)	7 (7.78)	59 (7.68)	0.893	/	/
NIs	20 (7.81)	14 (8.43)^a^	6 (6.67)^a,b^	19 (2.47)^b^	<0.001^***^	0.004^**^	3.117(1.425,6.820)
NHB	28 (10.94)	20 (12.05)^a^	8 (8.89)^a,b^	42 (5.47)^b^	0.003^**^	0.024^*^	2.055(1.098,3.843)
NA	12 (4.69)	9 (5.42)	3 (3.34)	46 (5.99)	0.435	/	/
Full-term neonates							
LBWI	5 (2.21)^a^	3 (2.07)^a,b^	2 (18.52)^b^	52(7.24)	0.006^**^	0.006^**^	0.231(0.082, 0.650)
NIs	13 (5.75)	9 (6.21)	4 (4.94)	19 (2.65)	0.041^*^	0.013^**^	2.622(1.223,5.621)
NHB	21 (9.29)	15 (10.34)	6 (7.41)	42 (5.85)	0.098	/	/
NA	5 (2.2)	3 (2.07)	2 (5.47)	46 (6.41)	0.015^*^	0.011^*^	0.293(0.114, 0.756)
Preterm neonates							
LBWI	16(53.33)^a^	11 (52.38)^a^	5 (55.56)^b^	7(14.00)	<0.001^***^	<0.001^***^	9.819(3.019, 31.936)
NIs	7 (23.33)^a^	5 (23.81)^a^	2 (22.22)^b^	0 (0.00)	<0.001^##***^	/^&^	/
NHB	7 (23.33)^a^	5 (23.81)^a^	2 (22.22)^b^	0 (0.00)	<0.001^##***^	/^&^	/
NA	7 (23.33)^a^	6 (28.57)^a^	1 (11.11)^b^	0 (0.00)	<0.001^##***^	/^&^	/

## 4 Discussion

Compared to the HC group, the most salient clinical manifestation in the OAPS group was pathological pregnancy, particularly a history of preterm labor, stillbirth, and miscarriages, all of which were significantly more frequent than in the HC group. The underlying causes of pathological pregnancy may involve activating endothelial cells, monocytes, and platelets by aPLs, leading to a procoagulant state. This process may be further exacerbated by the direct action of the placental trophoblast, resulting in trophoblast cell destruction and apoptosis. This process can lead to a cascade of adverse outcomes, including reduction of hormones such as human chorionic gonadotropin, diminished capacity of trophoblast cells to invade and implant in the uterus, and suppression of trophoblast cell proliferation. These alterations may hinder the embryo’s attachment to the uterus and progression of the pregnancy (16). Although the standard treatment regimen of low-dose aspirin combined with low-molecular-weight heparin significantly improved the live birth rate in patients with OAPS (17), our research found that, even after standard treatment, OAPS mothers and fetuses still face a series of significant residual risks, including a hypercoagulable state of the mother, unique placental pathological changes, and adverse neonatal outcomes. This finding indicates that the current therapy has limitations in correcting the fundamental pathophysiological mechanisms of OAPS, highlighting the urgency of assessing its impact on pregnancy quality beyond the live birth rate.

Key laboratory findings from this study delineate distinct hematological and biochemical profiles in OAPS patients compared to healthy controls. The OAPS group exhibited a significantly higher incidence of abnormalities in activated partial thromboplastin time (APTT) and prothrombin time (PT), yet a lower incidence of abnormal prothrombin activity (PTA). This apparent prolonged conventional coagulation time alongside a lower rate of reduced PTA likely reflects the therapeutic effect of low-molecular-weight heparin (LMWH) (18). The observed alterations in these coagulation parameters are consistent with the documented pharmacologic profile of LMWH calcium, as previously reported (19). LMWH, primarily through the inhibition of factor Xa, can induce a modest prolongation of APTT and PT, representing a predictable and intended anticoagulant state rather than a marker of bleeding risk (20). Concurrently, the significant elevations in the mean values and abnormality rates of D-dimer and fibrin degradation products (FDP) in the OAPS group provide direct laboratory evidence of ongoing fibrin formation and degradation, indicating a persistent hypercoagulable state with secondary fibrinolysis. Furthermore, the observed increase in total bile acid (TBA) levels suggests the presence of intrahepatic cholestasis in a subset of patients, which may compound obstetric risks. The higher incidence of red blood cell (RBC) and hemoglobin (HB) abnormalities is consistent with the greater prevalence of anemia observed in the OAPS cohort, implying a potential impact of the disease on hematopoiesis or erythrocyte stability. Collectively, these laboratory findings define a distinct hematological profile in OAPS, characterized by a treated prothrombotic phenotype and associated systemic involvement, underscoring the need for comprehensive monitoring.

The observed lower incidence of intrauterine distress in the OAPS group can be logically explained by its standardized clinical management. The implementation of intensified antenatal surveillance in these high-risk pregnancies facilitated timely detection of complications, leading to earlier elective delivery and thereby reducing the incidence of frank distress. In addition, a comprehensive review of the extant literature indicates that the effects of OAPS on fetal and neonatal outcomes primarily encompass birth weight, fetal death, preterm delivery, FGR, and fetal acidosis (21). In this study, the birth weight of neonates in the OAPS group was significantly lower than that in the HC group. However, there was no statistically significant difference in the proportion of low birth weight. Additionally, the OAPS group exhibited a higher proportion of preterm births than the HC group. Previous research found that 127 patients with OAPS were at risk of preterm labor, and the incidence of “spontaneous preterm labor” was 16.5% (22). Consequently, the occurrence of preterm labor in pregnancies affected by OAPS encompasses two distinct categories: (1) medically indicated preterm labor associated with pre-eclampsia or fetal factors and (2) a latent predisposition to preterm labor. The underlying predisposition to preterm labor is well-documented. Furthermore, the study noted that neonatal infections and hyperbilirubinemia were more prevalent in the OAPS group than in the HC group. Previous research has indicated that preterm infants are more susceptible to diseases and hyperbilirubinemia compared to term infants (23). To further investigate this observation, a subgroup analysis was conducted. Crucially, the subgroup analysis confirmed that this elevated risk remained significant even after accounting for prematurity, as the infection rate was also considerably higher in term neonates from the OAPS group. This suggests that the predisposition to infection is not solely a consequence of preterm birth but is intrinsically linked to the OAPS condition itself. We propose that the altered intrauterine environment in OAPS—characterized by a persistent placental inflammatory state and immune dysregulation (24)—may impair fetal immune system programming or function, increasing susceptibility to postnatal infection. These findings underscore that the ramifications of OAPS transcend the endpoint of live birth, warranting a shift in clinical focus from merely “securing fetal survival” to “safeguarding neonatal health.” This necessitates closer collaboration between obstetricians and neonatologists to optimize perinatal management from pregnancy into the postpartum period.

Given these persistent challenges in management, the recent introduction of the ACR/EULAR 2023 classification criteria for APS marks a significant step forward. These updated criteria refine the original Sydney standards by incorporating a weighted scoring system and expanded serological markers, thereby enhancing specificity for research (13). However, it is important to distinguish between classification criteria, designed to ensure cohort homogeneity, and the broader clinical spectrum of the disease. The stringent nature of such criteria may inevitably exclude some patients with highly suggestive clinical presentations—a group often identified as NC-OAPS. Our study found that there was no significant difference between the NC-OAPS group and the C-OAPS group, not only in obstetric history but also in terms of maternal complications, comorbidities, and outcomes (Tables 2–4). This key finding demonstrates that patients in the NC-OAPS group experience a clinical burden of disease comparable to that of patients who meet the formal criteria. By intentionally including NC-OAPS patients, our study aims to characterize the outcomes and pathophysiological features across the entire clinical phenotype managed as OAPS, thus providing insights that may inform future diagnostic and therapeutic strategies.

For patients diagnosed with OAPS, particularly those with recurrent pregnancy loss, combination therapy with low-dose aspirin and low-molecular-weight heparin remains the cornerstone of management and is effective in improving live birth rates (25). However, our findings underscore that this standard regimen does not fully eliminate significant residual risks, including maternal hypercoagulability, placental pathology, and adverse neonatal outcomes. Therefore, meticulous monitoring for maternal and neonatal complications is imperative, and management must be highly individualized. Future care for OAPS necessitates a paradigm shift, extending beyond anticoagulation. A proactive, multidisciplinary approach—involving obstetricians, rheumatologists, and often neonatologists—is essential throughout the pre-pregnancy, antenatal, and postpartum periods. The overarching goal must transition from merely “ensuring survival” to comprehensively “optimizing health.” Achieving this requires a dual strategy: advancing interventions to target fundamental pathophysiological mechanisms earlier in the disease course and deepening clinical vigilance toward placental function and long-term neonatal wellbeing. This redefined focus should guide the next generation of research and clinical innovation in OAPS.

## 5 Conclusion

This study, focusing on OAPS patients who achieved successful delivery, shows that despite standard treatment with low-dose aspirin and LMWH, these women continue to exhibit a distinct and complex clinical profile. Key findings include a persistent maternal hypercoagulable state, evidenced by elevated D-dimer and FDP and an increased risk of adverse neonatal outcomes, such as lower birth weight, preterm delivery, and a heightened susceptibility to neonatal infection and hyperbilirubinemia. These findings collectively indicate that the central clinical challenge in OAPS is shifting from achieving live birth to safeguarding the quality of pregnancy and neonatal health. Consequently, a paradigm shift in management is warranted—extending beyond current anticoagulation strategies to embrace a proactive, multidisciplinary approach focused on pre-pregnancy counseling, intensified placental and fetal surveillance, and long-term neonatal care, with the ultimate goal of optimizing overall maternal and offspring health outcomes.

## 6 Limitations

Several limitations of this study should be acknowledged. First, its retrospective and single-center design may introduce selection bias and limit the generalizability of the findings. The sample size, though substantial, may still be insufficient to detect significant differences in rarer complications. Second, the study population consisted of patients who ultimately achieved a successful delivery, which may not fully represent the entire spectrum of OAPS severity, particularly those with the majority of refractory cases. Furthermore, the absence of detailed placental histopathological analysis for all participants limits our ability to correlate clinical outcomes with specific underlying placental lesions. Third, the subgroup analyses based on antibody profiles were limited by small sample sizes, which may have obscured statistically significant differences and precluded a more robust comparison. Finally, potential unmeasured confounding factors, despite our matching efforts, could have influenced the observed associations. Future prospective, multi-center studies with larger cohorts and systematic placental examination are needed to validate these findings and further elucidate the pathophysiological mechanisms linking OAPS to adverse outcomes.

## Data Availability

The raw data supporting the conclusions of this article will be made available by the authors, without undue reservation.
