# Supplementary material for: Maternal and neonatal outcomes in obstetric antiphospholipid syndrome: a retrospective case-control study
Source: Front Med (Lausanne). 2025 Dec 10;12:1660134. doi: 10.3389/fmed.2025.1660134 (PMC12727887; doi:10.3389/fmed.2025.1660134)
Supplement: Supplementary file 1 [file Table_1.docx]

**Table A** Laboratory Classification of Typical OAPS Patients

| Laboratory classification |  | Classification and homotypic aPL | N(%) |
| --- | --- | --- | --- |
| I |  |  | 32(19.27) |
|  |  | Three positive | 3(1.81) |
|  |  | LA+ aCL IgM+ aCL IgG+ antiβ2GPI-IgM+ anti β2GPI-IgG | 3(1.81) |
|  |  | Double positive | 29(17.47) |
|  |  | LA + anti β2GPI-IgG | 8(4.82) |
|  |  | LA+ anti β2GPI-IgM | 8(4.82) |
|  |  | LA+ aCL IgM | 10(6.02) |
|  |  | aCL IgM+ aCL IgG+ anti β2GPI-IgM+ anti β2GPI-IgG | 1(0.60) |
|  |  | LA+aCL IgM+ aCL IgG | 2(1.20) |
| II |  |  | 134(80.72) |
|  | IIa | LA | 89(53.61) |
|  | IIb |  | 26(15.66) |
|  |  | aCL IgM | 8(4.82) |
|  |  | aCL IgG | 3(1.81) |
|  |  | aCL IgM+ aCL IgG | 15(9.04) |
|  | IIc |  | 19(11.45) |
|  |  | anti β2GPI-IgM | 12(7.23) |
|  |  | anti β2GPI-IgG | 1(0.60) |
|  |  | anti β2GPI-IgM+anti β2GPI-IgG | 6(3.61) |
